# Transfusion practices, thresholds, and complications in pediatric oncology patients: a single-center prospective longitudinal observational study

**DOI:** 10.3389/fped.2026.1955442

**Published:** 2026-09-30

**Authors:** Büşra Tunalı Özcan, Sonay İncesoy Özdemir, Handan Dinçaslan, Nihal Ekin Dağ, Cem Çanakçi, Vafa Maharramova, Nübar Mustafayeva, Emel Ünal, Nurdan Taçyıldız

**Affiliations:** 1 Department of Pediatrics, Ankara University Faculty of Medicine, Ankara, Türkiye; 2 Department of Pediatrics, Division of Pediatric Oncology, Ankara University Faculty of Medicine, Ankara, Türkiye

**Keywords:** blood transfusion, childhood cancers, platelet transfusion, red blood cell transfusion, transfusion reaction, transfusion threshold

## Abstract

This study aimed to evaluate the frequency, indications, pre-transfusion threshold values, associated risk factors, and complications of blood product transfusions in pediatric oncology patients to contribute to the development of evidence-based, standardized transfusion strategies. This single-center, prospective observational study included 41 pediatric oncology patients aged 1 to 18 years who received red blood cell (RBC), platelet, or fresh frozen plasma (FFP) transfusions between June 2023 and June 2024. Patients were followed throughout their treatment and for six months after their final transfusion. Among 404 transfusions (215 RBC, 183 platelet, 6 FFP), the mean number per patient was 9.78 ± 9.2. Pre-transfusion platelet thresholds were significantly higher in the leukemia/lymphoma group than in the solid tumor group (23,892 ± 11,773/mm^3^ vs. 16,669 ± 11,921/mm^3^; *p* < 0.001), while RBC hemoglobin thresholds were similar (7.4 ± 0.6 vs. 7.3 ± 0.8 g/dL; *p* = 0.67). Advanced-stage disease (*p* = 0.04) and relapsed or refractory disease (*p* = 0.026) were associated with increased RBC transfusion requirements. Acute reactions occurred in 12.4% of transfusions, most (84%) being febrile nonhemolytic reactions. Premedication did not prevent reactions (*p* = 0.6). During long-term follow-up, serum ferritin exceeded 1000 ng/mL in 45.7% of patients. Transfusion management varies by diagnosis, with platelet thresholds in hematologic malignancies maintained higher than literature recommended. Advanced-stage and relapsed or refractory disease increase the transfusion burden. The high incidence of acute reactions and iron overload underscores the need for transfusion safety, long-term monitoring, and standardized, evidence-based transfusion strategies in pediatric oncology.

## 1 Introduction

Diagnostic and therapeutic advances over the past three decades have significantly improved survival rates for childhood cancers (1). Currently, long-term survival rates for many pediatric malignancies exceed 80%. This success is due not only to effective antineoplastic therapies but also to comprehensive supportive care, including infection control, nutritional support, and transfusion practices (2). Intensive chemotherapy protocols, hematopoietic stem cell transplantation procedures, and disease-related bone marrow suppression have made red blood cell (RBC) and platelet transfusions essential components of treatment for childhood cancers (3). Although blood product transfusions are life-saving interventions for correcting symptomatic anemia, preventing hemorrhage, and ensuring continuity of treatment, they are associated with various short- and long-term complications (4–6). Early complications include febrile non-hemolytic reactions, allergic reactions, acute hemolytic reactions, transfusion-related acute lung injury, alloimmunization, and risk of infection. Late complications, such as iron overload and organ toxicities, pose significant clinical challenges, particularly in patients receiving multiple RBC transfusions (7). With increasing survival rates in childhood cancers, transfusion safety and long-term transfusion outcomes have become increasingly important.

In recent years, restrictive transfusion strategies have gained prominence to reduce unnecessary transfusions, and numerous adult studies have demonstrated the safety of lower hemoglobin (Hb) thresholds (8). Similarly, the efficacy of lower threshold values for prophylactic platelet transfusions is well supported (9, 10). However, much of the current guidance is based on adult studies, and direct extrapolation to pediatric oncology patients is not always appropriate. Due to age-related physiological differences, the distribution of malignancy types, specific treatment protocols, and variability in bleeding risk profiles among children mean that transfusion decisions are often individualized and influenced by clinician experience (11). Indeed, despite the existence of international guidelines, significant variations between centers in transfusion thresholds and clinical approaches have been reported (12). Standardized practices have not yet been fully established for issues such as platelet transfusion thresholds in hematologic malignancies, strategies during episodes of febrile neutropenia, and the routine use of premedication. Furthermore, real-world data that simultaneously evaluate transfusion frequency, threshold values, risk factors, and complications in pediatric oncology patients are limited. Studies on this topic in our country are particularly scarce, highlighting the need for comprehensive observational data reflecting current practices.

The aim of this study is to evaluate the frequency, indications, and threshold values for RBC, platelet, and fresh frozen plasma (FFP) transfusions administered to pediatric cancer patients; to identify clinical factors affecting transfusion requirements; and to investigate early and late transfusion-related complications. These findings are expected to contribute to the development of evidence-based, standardized transfusion strategies for pediatric oncology patients.

## 2 Materials and methods

### 2.1 Study design and patient selection

The study protocol was approved by the Ankara University Faculty of Medicine Human Research Ethics Committee (Decision No. İ06-361-23), and written informed consent was obtained from the parents or legal guardians of all participants. This single-center observational study was conducted at the Division of Pediatric Oncology, Ankara University Faculty of Medicine, from 15 June 2023 to 15 June 2024. The study enrolled 41 patients aged 1 to 18 years with a confirmed cancer diagnosis who received at least one transfusion of RBCs, platelets, or FFP. Patients with concomitant hematological disorders requiring chronic transfusion support (e.g., thalassemia) were excluded.

### 2.2 Data collection and transfusion protocol

Patient demographics, diagnoses, disease stages, and treatment protocols were recorded using standardized data forms. For each transfusion episode, the clinical indication (e.g., prophylaxis, febrile neutropenia, active bleeding), pre-transfusion Hb and platelet counts, and any complications occurring within 24 hours post-transfusion were monitored. To evaluate iron overload as a late complication, serum ferritin levels at the six-month follow-up were assessed. Since ferritin is an acute phase reactant, C-reactive protein (CRP) levels were evaluated concurrently. Ferritin levels from patients with concurrent infections and elevated CRP values were excluded from the analysis. In accordance with institutional protocol, all administered blood products were irradiated and leukoreduced. RBCs were transfused at a dose of 10–15 mL/kg over a 4-hour period and platelets were administered at a dose of 10–15 mL/kg over a 2-hour period.

According to our institutional guidelines, Hb threshold for RBC transfusions in chemotherapy patients without additional complications is generally 7–8 g/dL. Transfusion decisions are highly individualized based on patient characteristics; for asymptomatic patients, transfusions are typically administered at Hb levels between 6 and 7 g/dL. For platelet transfusions, the recommended prophylactic threshold is ≤10,000/mm^3^. However, higher threshold levels are utilized for patients presenting with active bleeding, high fever, hyperleukocytosis, a rapid decline in platelet counts, coagulation disorders, or those undergoing invasive procedures (13).

In accordance with national blood banking regulations and our institutional protocol, RBC units are prepared with SAG-M (saline, adenine, glucose, mannitol) additive solution and have a maximum storage duration of 42 days. Platelet concentrates are primarily prepared in plasma and can be stored for a maximum of 5 days. During the study period, routine washing of RBC or platelet units, as well as specific pathogen reduction technologies for platelets, were not standard practices at our center. Instead, to ensure transfusion safety, all administered blood products were universally leukoreduced and subjected to gamma irradiation (13). Routine premedication was not administered prior to transfusions, and there is no standardized protocol for premedication at our institution. The decision to administer premedication was made on an individual patient basis, with paracetamol and antihistamines being the most frequently utilized agents.

Among the transfusion reactions observed in our study, a febrile non-hemolytic transfusion reaction (FNHTR) was defined as an unexplained rise in body temperature of ≥ 1°C reaching ≥ 38°C during or within 4 hours after the completion of the transfusion (14). Immune hemolytic transfusion reactions (IHTR) were identified by the sudden onset of fever, chills, flank pain, or hypotension within the first 24 hours following transfusion (15). Allergic and anaphylactic reactions were classified based on clinical symptoms ranging from isolated urticaria to severe respiratory distress and hypotension occurring within 4 hours of transfusion initiation (16). Hypotensive reactions were characterized by a sudden decline in blood pressure during the initial minutes of transfusion that rapidly resolved upon cessation of the infusion (17). Post-transfusion purpura (PTP) was identified by profound thrombocytopenia and bleeding, typically emerging five to ten days post-transfusion (18).

### 2.3 Statistical analysis

Data were analyzed using the Statistical Package for the Social Sciences (SPSS) software. In our study, gender, diagnosis and diagnostic group, disease stage and risk group, relapsed or refractory status, administered treatments, survival status, type of blood product, transfusion indications, premedication use, and types of transfusion reactions were considered categorical variables; whereas age at diagnosis, baseline hemoglobin and platelet counts, pre-transfusion hemoglobin and platelet thresholds, number of transfusions per patient, and serum ferritin levels were defined as continuous variables. Categorical variables were compared using the chi-square test, while continuous variables were analyzed using the Student's t-test, Mann–Whitney U test, or Kruskal–Wallis test, depending on the normality of their distribution and the number of groups compared. Statistical significance was defined as a *p*-value <0.05.

## 3 Results

### 3.1 Patient characteristics

A total of 41 pediatric oncology patients were included in the study. The mean age of the cohort was 8.3 ± 5.3 years, and 22 patients (53.7%) were male. Twenty-seven patients (65.9%) were diagnosed with solid tumors, and 14 patients (34.1%) had hematological malignancies (leukemia or lymphoma).

Given the heterogeneity of the underlying malignancies, disease extent was characterized using diagnosis-specific staging or risk classification systems. Among patients with stage-classifiable malignancies, 6 (14.6%), 8 (19.5%), and 7 (17.1%) patients were classified as stage II, III, and IV disease, respectively. In patients with acute lymphoblastic leukemia, one patient was classified as standard-risk and four as high-risk according to disease-specific risk stratification criteria. Among patients with solid tumors, five patients (12.2%) had metastatic disease and ten (24.4%) had localized disease at diagnosis. Bone marrow involvement was present in 10 patients (24.4%).

All patients received multiagent chemotherapy; 23 patients (56.1%) additionally received radiotherapy and 28 patients (68.3%) underwent surgical treatment. At the end of follow-up, 27 patients (65.9%) were alive and in remission, whereas 14 patients (34.1%) had died due to relapsed or refractory disease. Baseline demographic and clinical characteristics are summarized in Table 1.

**Table 1:** Demographic, clinical, and laboratory characteristics of the patients (*n* = 41).

Characteristics	Subgroup/value	Number (*n*)	Percentage (%)
Gender	Female	19	46.3
	Male	22	53.7
Age at diagnosis (years)	Mean ± SD	8.3 ± 5.3	–
Diagnosis group	Solid Tumors	27	65.9
	Leukemia and Lymphoma	14	34.1
Diagnosis	Leukemia	5	12.2
	Lymphoma	9	22.0
	CNS Tumors	7	17.1
	Sympathetic Nervous System Tumors	3	7.3
	Retinoblastoma	1	2.4
	Kidney Tumors	1	2.4
	Liver Tumors	2	4.9
	Bone Tumors	8	19.5
	Soft Tissue Sarcomas	3	7.3
	Epithelial Tumors/Melanoma	1	2.4
	Other	1	2.4
Risk-stage	Standard Risk	1	2.4
	High Risk	4	9.8
	Stage 2	6	14.6
	Stage 3	8	19.5
	Stage 4	7	17.1
	Metastatic	5	12.2
	Non-Metastatic	10	24.4
Administered treatments	Chemotherapy	41	100.0
	Surgery	28	68.3
	Radiotherapy	23	56.1
Baseline laboratory values	Hemoglobin (g/dL) (Mean ± SD)	11.4 ± 2.3	–
	Platelet (/mm^3^) (Mean ± SD)	361,097 ± 169,179	–

### 3.2 Transfusion characteristics

During the study period, a total of 404 transfusion episodes were recorded, including 215 RBC transfusions, 183 platelet transfusions, and 6 FFP transfusions. Forty of the 41 patients received RBC transfusions, whereas 28 patients received platelet transfusions. The median number of transfusions per patient was 6 (range, 1-34) units. The most common indication for both RBC (36.7%) and platelet (55.2%) transfusions was febrile neutropenia, followed by prophylactic transfusions (Table 2).

**Table 2:** Transfusion frequency and distribution of indications.

Transfusion characteristics	Red blood cell (RBC)	Platelet (PLT)	Fresh frozen plasma (FFP)
Median number per patient (range)	4 (1–18)	4 (1–26)	1 (1–2)
Total number of transfusions (*n*)	215	183	6
Indications *n* (%)			
Febrile neutropenia	79 (36.7%)	101 (55.2%)	–
Prophylactic	78 (36.3%)	37 (20.2%)	–
Sepsis	22 (10.2%)	10 (5.5%)	–
Postoperative	11 (5.1%)	2 (1.1%)	4 (66.7%)
Pneumonia with concurrent (hypoxemia/respiratory distress)	6 (2.8%)	14 (7.7%)	–
Radiotherapy period	6 (2.8%)	9 (4.9%)	–
Prior to invasive procedure	4 (1.9%)	3 (1.6%)	1 (16.7%)
Bleeding/other	9 (4.2%)	7 (3.8%)	1 (16.7%)

### 3.3 Transfusion thresholds according to disease group

Mean pre-transfusion hemoglobin thresholds for RBC transfusions were similar between the leukemia/lymphoma and solid tumor groups (7.4 vs. 7.3 g/dL; *p* = 0.67).

In contrast, the mean pre-transfusion platelet thresholds were significantly higher in the leukemia/lymphoma group (23,892 ± 11,773/mm^3^) compared to the solid tumor group (16,669 ± 11,921/mm^3^) (*p* < 0.001) (Table 3).

**Table 3:** Pre-transfusion threshold values by diagnosis group.

Variables	Leukemia and lymphoma (*n* = 14)	Solid tumors (*n* = 27)	*p*-Value
Hemoglobin threshold (g/dL)			
Mean ± SD	7.4 ± 0.6	7.3 ± 0.8	0.67
Median (Min-Max)	7.4 (6.2–10.1)	7.5 (5.5–10.3)	
Platelet threshold (/mm^3^)			
Mean ± SD	23,892 ± 11,773	16,669 ± 11,921	**<0.001**
Median (Min-Max)	22,500 (5,000–59,000)	15,000 (2,000–62,000)	

Bold values indicate statistically significant *p*-values (*p* < 0.05).

### 3.4 Factors associated with transfusion burden

Analysis of factors affecting transfusion burden showed that age, sex, and diagnostic group did not significantly affect the total number of transfusions. However, advanced-stage disease and relapsed/refractory disease status were significantly associated with increased RBC transfusion burden (*p* = 0.04 and *p* = 0.026, respectively). Transfusion burden by risk factors is shown in Tables 4, 5.

**Table 4:** Comparison of transfusion burden by patient risk factors.

Variables	*n*	RBC median (range)	*p*	*n*	PLT median (range)	*p*
Standard risk	1	4	1^a^	1	2	0.5^a^
High risk	4	6.5 (2–8)		3	6 (3–20)	
Stage 2	5	1 (1–3)	**0.04** ^b^	3	2 (2–3)	0.27^b^
Stage 3	8	6 (1–18)		6	4.5 (1–14)	
Stage 4	7	3 (1–17)		3	4 (3–19)	
Metastatic	5	5 (4–17)	0.76^a^	4	8 (1–26)	0.68^a^
Non-metastatic	10	5.5 (1–12)		8	4.5 (1–14)	

a Evaluated using the Mann–Whitney U test for comparisons between two independent groups (Standard vs. High Risk, and Metastatic vs. Non-Metastatic).

b Evaluated using the Kruskal–Wallis test for comparisons across multiple disease stages (Stage 2 vs. Stage 3 vs. Stage 4); a statistically significant difference was found between Stage 2 and Stage 3.

Bold values indicate statistically significant *p*-values (*p* < 0.05).

**Table 5:** Comparison of transfusion burden by relapsed or refractory disease status.

Relapsed/Refractory Disease	*n*	RBC median (range)	*p*	*n*	PLT median (range)	*p*
Present	15	5 (2–18)	**0.026**	13	4 (1–26)	0.23
Absent	25	3 (1–10)		15	4 (1–11)	

Bold values indicate statistically significant *p*-values (*p* < 0.05).

### 3.5 Transfusion reactions and long-term complications

Acute transfusion reactions occurred in 50 (12.4%) of the 404 transfusions administered. Of the 50 transfusions that developed a reaction, 21 (42%) were RBC transfusions and 29 (58%) were platelet transfusions. When evaluated by reaction type, the 21 RBC-associated adverse events consisted of 20 cases of FNHTR and 1 IHTR. The 29 platelet-associated adverse events included 22 cases of FNHTR, 5 cases of hypotension, 1 mild allergic reaction, and 1 case of PTP.

The most frequently observed reaction was FNHTR, accounting for 84% of all acute events (Table 6). No statistically significant difference in the incidence of reactions was observed between transfusions administered with premedication (24.5%) and those without premedication (*p* = 0.6).

**Table 6:** Transfusion reactions and complications.

Reaction type	Total number *n* = 50 (%)	Associated with RBC *n* = 21 (42%)	Associated with platelet *n* = 29 (58%)
Febrile non-hemolytic (FNHTR)	42 (84%)	20	22
Hypotension	5 (10%)	–	5
Immune hemolytic transfusion reaction (IHTR)	1 (2%)	1	–
Post-transfusion purpura (PTP)	1 (2%)	–	1
Mild allergic reaction	1 (2%)	–	1

During long-term follow-up, serum ferritin levels exceeded 1000 ng/mL in 45.7% of patients. The median ferritin level in our cohort was 900 ng/mL (range, 44.4–9506 ng/mL). Out of 41 patients (accounting for a total of 404 transfusion episodes), only 1 patient received exclusively platelet transfusions without any RBC support. Consequently, comparing ferritin levels between patients receiving RBC transfusions and those receiving exclusively platelet transfusions was neither statistically feasible nor clinically meaningful.

## 4 Discussion

This study presents real-world data that comprehensively evaluate transfusion practices, threshold values, associated risk factors, and complications in pediatric oncology patients. To our knowledge, this is one of the first studies to longitudinally investigate transfusion practices among pediatric cancer patients in our country. Another notable strength is the provision of real-world data from a tertiary referral center in a middle-income country. Most existing literature on pediatric transfusion practices predominantly comes from high-income countries (such as the multicenter cohort studies conducted by Goel et al. and Nellis et al.) (4, 10), while data on how these practices are shaped across different healthcare systems remain limited. Transfusion strategies may vary substantially among countries due to differences in resource accessibility, blood product availability, hemovigilance practices, and clinical decision-making processes. Consequently, our findings not only reflect national practices but also contribute to the international literature on transfusion management for pediatric oncology patients in middle-income settings. Our results show that transfusion requirements vary according to disease type and stage, and that platelet transfusion practices, particularly in hematologic malignancies, are more liberal than current guideline recommendations (such as those established by the AABB and ASCO) (19, 20).

In our cohort, the median number of transfusions per patient was 6 (range, 1–34), which appears higher than those reported in several other series in the literature (21). The most plausible explanation for this discrepancy is that a substantial proportion of our patient cohort consisted of advanced-stage, high-risk, and relapsed or refractory cases. Indeed, our analyses demonstrated that advanced-stage disease and relapsed or refractory status significantly increased the requirement for RBC transfusions. Previous studies have also reported that the need for supportive care is markedly elevated in patients receiving intensive chemotherapy and presenting with a high disease burden (22).

Both RBC and platelet transfusions were most frequently administered during episodes of febrile neutropenia. During febrile neutropenia, the infection-induced inflammatory response, exacerbated bone marrow suppression, and increased platelet consumption can collectively increase transfusion requirements. However, current guidelines emphasize that transfusion decisions should not be based solely on the presence of fever, but must be evaluated in conjunction with the patient's overall clinical condition and bleeding risk (19). Furthermore, regarding the transfusion indications detailed in our cohort, conditions such as pneumonia are not considered standalone transfusion indications per se; rather, this category comprises patients who experienced acute pulmonary infections complicated by concurrent clinical deteriorations—such as respiratory distress, hypoxemia, or symptomatic anemia—that necessitated supportive transfusion therapy. Consequently, transfusion decisions for these patients were guided by the overall clinical status and physiological requirements rather than the pulmonary infection itself.

One of the most significant findings of our study is that the pre-transfusion platelet threshold in the leukemia/lymphoma group was significantly higher than in the solid tumor group. The British Society for Haematology and other international guidelines generally recommend a threshold of 10,000/mm^3^ for prophylactic platelet transfusions in hematology-oncology patients without active bleeding (23). Despite these recommendations, the mean platelet threshold for hematologic malignancies in our study was approximately 24,000/mm^3^. This finding should be re-evaluated within the context of the patients’ acute clinical conditions. In our cohort, febrile neutropenia appears to be one of the most common clinical indications associated with platelet transfusion. In the presence of fever, infection, or other additional bleeding-risk factors, a threshold higher than the standard 10,000/mm^3^ used for otherwise stable patients may be clinically reasonable. Similarly, previous observational studies have reported that clinicians often prefer higher thresholds, particularly for patients with acute leukemia, due to the perceived risk of bleeding (20, 24). However, current evidence shows that more liberal platelet transfusion strategies do not significantly reduce the incidence of major hemorrhage (24). This finding underscores the critical need for standardized transfusion practices in pediatric oncology. In contrast, similar hemoglobin thresholds were used for RBC transfusions in both patient groups. The mean threshold values of 7.0–7.5 g/dL align with the TAXI consensus recommendations and restrictive RBC transfusion strategies (25). Recent data indicate that this approach is safe in hemodynamically stable pediatric patients and can effectively reduce unnecessary transfusion exposure (26, 27).

When evaluated for transfusion safety, the rate of acute reactions was 12.4%. Although this rate is higher than that reported in some previous studies (such as the retrospective investigations by Yanagisawa et al. and Moncharmont et al., which reported reaction rates of 3.6% and 6.5%, respectively), the vast majority of these reactions were FNHTRs, and severe reactions were rare (28, 29). One plausible explanation for the relatively high incidence of reactions is the prospective and longitudinal design of our study. Standardized monitoring of all transfusion episodes and active surveillance likely increased detection, particularly of mild reactions. In contrast, many studies in the literature rely on retrospective chart reviews and may be subject to underreporting bias. It is well established that active hemovigilance systems detect higher reaction rates than passive reporting methods (30).

Evaluating the rates of FNHTR, which accounted for the majority of acute reactions in our study, presents a clinical challenge. Since many of our patients received transfusions for febrile neutropenia, their baseline temperatures were already elevated. As a result, it is difficult to definitively determine whether a fever around the time of transfusion is a direct reaction to the blood product or a consequence of the underlying disease or an existing infection. To manage this diagnostic difficulty, temperatures were closely monitored during and after the transfusions, and the clinical status of the patients was evaluated comprehensively alongside other infection markers.

The observation that premedication did not reduce the incidence of transfusion reactions is noteworthy. Systematic reviews and meta-analyses show that routine administration of acetaminophen or antihistamines does not significantly decrease the occurrence of febrile or allergic reactions (31). Consistent with this, our findings suggest that individualized approaches tailored to specific patient cohorts may be more appropriate than routine premedication.

The finding that serum ferritin levels exceeded 1000 ng/mL in about half of the patients during long-term follow-up indicates that transfusion-related iron overload remains a significant clinical challenge. As survival rates for childhood cancers continue to rise, late complications have become increasingly prominent. In patients receiving multiple RBC transfusions, monitoring ferritin levels, assessing organ iron burden, and initiating chelation therapy when necessary must be integral parts of long-term follow-up (32).

This study has several limitations. Its single-center design and relatively small sample size may limit the generalizability of the findings. Furthermore, although the transfusion requirements of these two disease groups may differ significantly, patients with leukemia (*n* = 5) and lymphoma (*n* = 9) were grouped together for statistical analyses due to this limited sample size. Consequently, their combined analysis may mask disease-specific differences. Additionally, because some transfusion decisions depend on individual clinician preference, variability in clinical practice is inevitable. Despite these limitations, the comprehensive evaluation of all transfusion episodes, inclusion of patients with both hematologic malignancies and solid tumors, and simultaneous assessment of acute and late complications within a single cohort are major strengths of this study.

## 5 Conclusion

Transfusion practices in pediatric oncology patients vary according to disease type, stage, and clinical condition. While restrictive red blood cell transfusion strategies are recommended by current clinical guidelines, their actual implementation in routine clinical practice may vary, and platelet transfusion thresholds in patients with hematologic malignancies remain higher than those recommended by current international guidelines, indicating persistent variation in clinical practice. Advanced-stage and relapsed/refractory disease were associated with increased transfusion requirements, whereas the relatively high rate of acute transfusion reactions and frequent iron overload emphasize the importance of both transfusion safety and long-term monitoring.

These findings support the need for standardized, evidence-based transfusion strategies tailored to pediatric cancer patients. Larger multicenter prospective studies, particularly those providing real-world data from middle-income countries, are needed to refine transfusion thresholds and optimize supportive care practices in pediatric oncology.

## Data Availability

The raw data supporting the conclusions of this article will be made available by the authors, without undue reservation.
